# Supplementary material for: Microplastics in Widely Used Polypropylene-Made Food Containers
Source: Toxics. 2022 Dec 7;10(12):762. doi: 10.3390/toxics10120762 (PMC9786867; doi:10.3390/toxics10120762)
Supplement: Supplementary file 1 [file toxics-10-00762-s001.zip › toxics-1974337-supplementary.pdf]

SUPPORTING INFORMATION

Microplastics in Widely Used Polypropylene-Made Food Containers

Jun Hu [1,2], Xin Xu [1], Ying Song [3], Wenqi Liu [4], Jianqiang Zhu [1], Hangbiao Jin [1,*], Zhu Meng [1]

[1]College of Environment, Zhejiang University of Technology, Hangzhou 310032, China;

[2]Innovation Research Center of Advanced Environmental Technology, Eco-Industrial Innovation Institute ZJUT, Quzhou 324018, China

[3]Quzhou Municipal Bureau of Ecology and Environment, Quzhou 324007, China

[4]Shaoxing Industrial Science Design Research Institute Co., Ltd. Hangzhou Branch, Hangzhou 310052, China

Table S1. Information on Sampling Sites and Materials of Take-Out Food Containers.

	Sampling site		*n*	Recycling symbol	Identification code
**Hangzhou (*n* = 35)**	Shangcheng District	6 restaurants	10	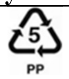	JQ-500;<br>B-T2;<br>#1422;<br>1RR-N
	Xihu District	9 restaurants	14		
	Binjiang District	6 restaurants	7		
	Gongshu District	3 restaurants	4		
**Xining (*n* = 37)**	Chengdong District	7 restaurants	13	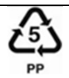	2B3J;<br>JA-100;<br>JB-B2
	Chengxi District	9 restaurants	12		
	Chengbei District	7 restaurants	12		
**Dalian (*n* = 32)**	Zhongshan District	8 restaurants	10	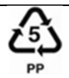	A-10-B;<br>T-6;<br>G01
	Xigang District	8 restaurants	9		
	Shahekou District	6 restaurants	13		
**Qingdao (*n* = 28)**	Laoshan District	5 restaurants	11	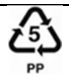	0FFF;<br>La-4;<br>JBC
	Shibei District	6 restaurants	10		
	Licang District	6 restaurants	7		
**Guangzhou (*n* = 21)**	Baiyun District	4 restaurants	5	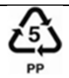	Qsd-3-1;<br>002;<br>B-T2;<br>24BD
	Tianhe District	5 restaurants	6		
	Yuexiu District	6 restaurants	6		
	Huadu District	2 restaurants	4		
**Chengdu (*n* = 27)**	Jinniu District	4 restaurants	6	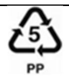	230;<br>ID-D;<br>JA-100;<br>9W2
	Qingyang District	4 restaurants	6		
	Wuhou District	3 restaurants	7		
	Gaoxin District	5 restaurants	8		
**Shanghai (*n* = 30)**	Huangpu District	5 restaurants	10	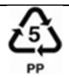	QQ-LID;<br>G1-0-f;<br>US-78;<br>H-22JB
	Xuhui District	7 restaurants	10		
	Pudong District	2 restaurants	4		
	Hongkou District	5 restaurants	6		

Table S2. Percentage (%; Mean, Range) of Different Shape, Size, Color, and Composition of Microplastics in Take-Out Food Containers Collected from Chinese Cities.

Shape	Chengdu	Xining	Dalian	Qingdao	Guangzhou	Hangzhou	Shanghai
**Fiber**	75<br>63–81	83<br>73–90	71<br>60–84	87<br>59–91	76<br>56–88	66<br>59–72	68<br>50–84
**Fragment**	18<br>10–26	14<br>8.1–20	23<br>11–30	6.2<br>4.2–9.3	17<br>12–24	28<br>20–37	25<br>10–31
**Film**	7.1<br>4.6–9.1	3.7<br>1.3–5.0	6.3<br>3.5–9.6	6.5<br>4.7–10	7.2<br>4.9–9.5	5.7<br>3.6–9.4	6.9<br>5.3–8.7
Size (μm)	**Chengdu**	**Xining**	**Dalian**	**Qingdao**	**Guangzhou**	**Hangzhou**	**Shanghai**
**50–100**	12<br>7.5–17	14<br>10–18	9.9<br>6.5–13	11<br>8.4–15	12<br>7.9–16	8.7<br>4.4–13	9.6<br>6.0–13
**101–200**	20<br>14–25	22<br>19–24	17<br>13–22	30<br>15–34	19<br>12–23	22<br>13–24	30<br>25–34
**201–500**	42<br>33–50	42<br>36–41	37<br>30–45	26<br>22–31	37<br>34–41	37<br>34–42	34<br>30–39
**501–1000**	23<br>17–34	16<br>12–19	25<br>17–29	25<br>22–29	23<br>19–28	21<br>17–25	18<br>13–22
**> 1000**	3.9<br>2.0–5.6	6.4<br>4.7–8.5	13<br>10–17	7.7<br>4.8–9.0	10<br>7.9–13	11<br>8.7–15	8.7<br>5.3–11
Color	**Guangzhou**	**Hangzhou**	**Chengdu**	**Dalian**	**Shanghai**	**Xining**	**Qingdao**
**Transparent**	65<br>60–71	73<br>68–77	41<br>35–45	56<br>51–62	70<br>61–74	42<br>39–46	39<br>34–43
**White**	21<br>17–24	22<br>17–26	20<br>15–24	17<br>14–22	23<br>20–27	12<br>10–15	29<br>24–36
**Blue**	9.1<br>6.6–14	1.2<br>0.47–2.6	11<br>6.9–15	17<br>13–20	1.1<br>0.56–1.6	15<br>12–17	9.3<br>6.4–15
**Black**	3.8<br>1.9–5.5	3.6<br>2.0–5.2	20<br>12–23	8.3<br>6.1–11	4.0<br>1.9–5.9	22<br>16–24	18<br>12–28
**Yellow**	1.4<br>0.87–1.6	0<br>0	7.4<br>4.2–11	3.2<br>1.9–5.2	1.6<br>0.88–3.3	8.6<br>4.7–11	5.2<br>3.5–7.0
Composition	**Guangzhou**	**Hangzhou**	**Chengdu**	**Dalian**	**Shanghai**	**Xining**	**Qingdao**
**PP**	65<br>61–70	73<br>70–75	61<br>58–66	56<br>52–64	70<br>66–75	72<br>67–78	59<br>55–65
**PE**	15<br>12–19	19<br>15–24	5.5<br>4.1–7.8	6.4<br>4.4–8.0	20<br>17–22	4.8<br>2.9–6.3	12<br>10–14
**Polyester**	8.9<br>6.1–11	1.5<br>0.50–2.6	9.4<br>5.3–15	17<br>12–21	1.1<br>0.46–1.7	5.1<br>2.6–8.1	9.9<br>6.4–13
**Polystyrene**	3.4<br>1.0–5.5	4.8<br>2.5–7.8	7.6<br>3.9–11	8.2<br>4.7–13	4.5<br>2.2–6.4	3.4<br>2.6–4.0	3.0<br>1.2–4.4
**polyurethane**	2.6<br>1.1–4.2	0<br>0	8.2<br>4.9–11	3.0<br>1.1–4.7	1.8<br>1.0–2.4	9.3<br>5.7–11	5.3<br>2.8–7.7
**Others**	4.7<br>2.3–6.6	1.7<br>1.0–2.3	8.1<br>4.7–14	9.3<br>6.6–15	2.4<br>1.0–4.1	5.5<br>2.5–7.9	11<br>8.5–16

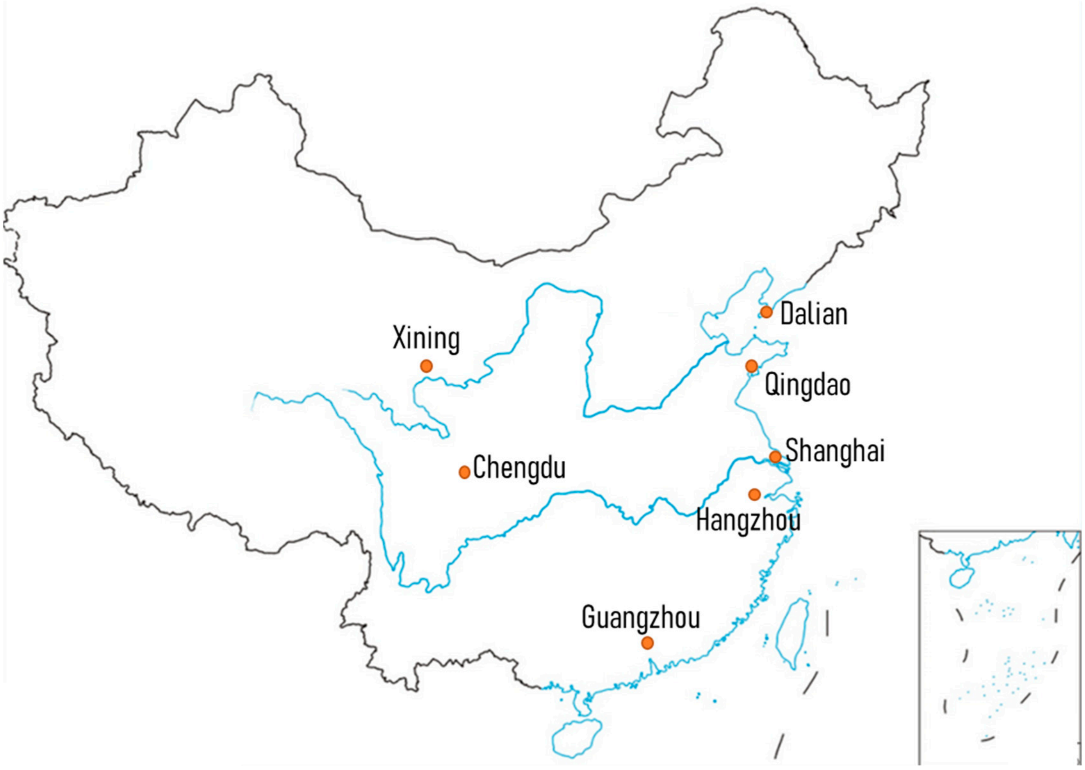

Figure S1. The map of sampling areas in China, including Chengdu, Xining, Dalian, Qingdao, Guangzhou, Hangzhou, and Shanghai.

(A)

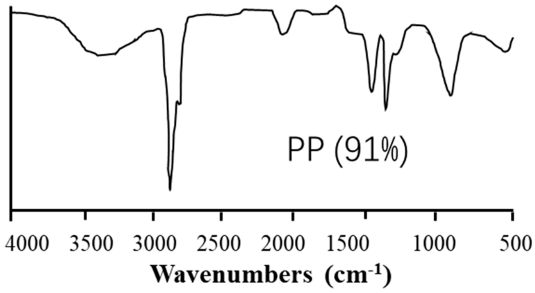

(B)

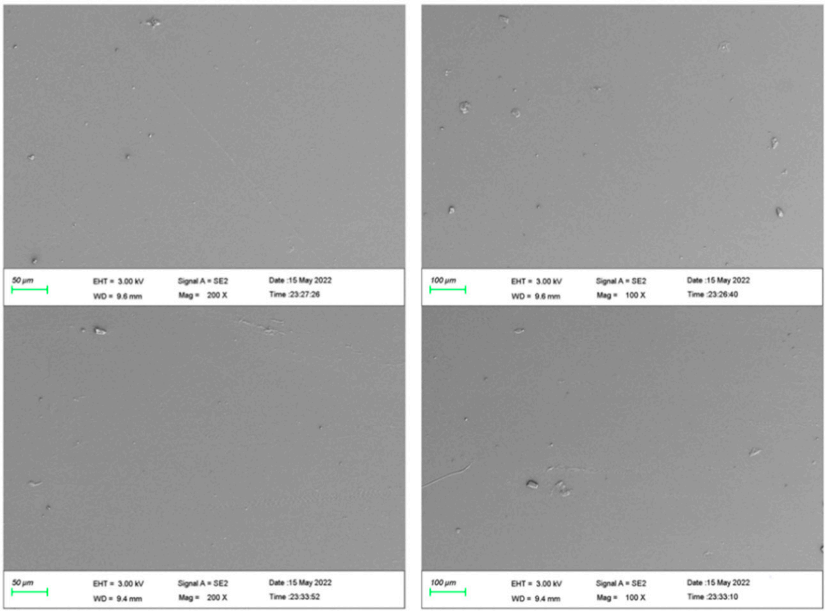

(C)

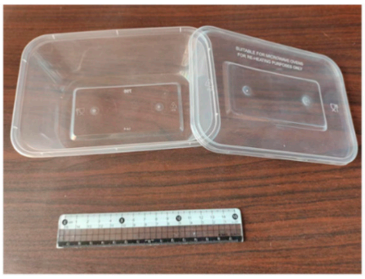

Figure S2. (A) A typical FT-IR spectrum of a TOFC sample. (B) SEM images of the inner surface of TOFCs. (C) A typical TOFC sample collected in this study.

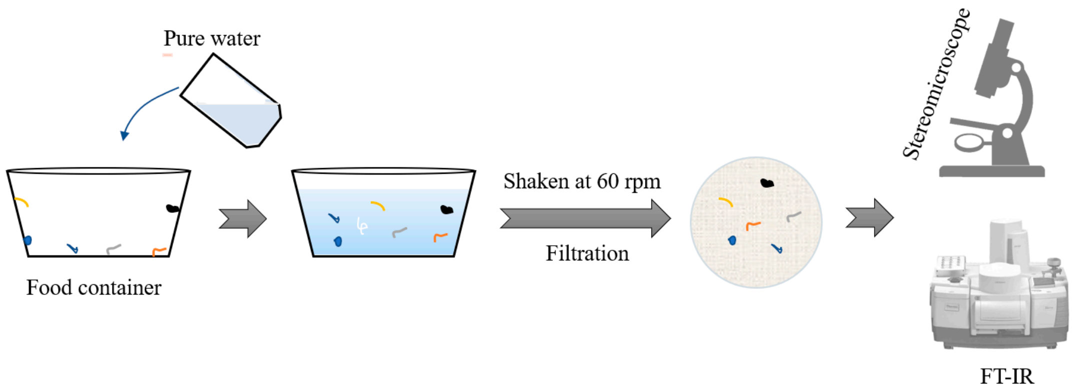

Figure S3. The TOFC sample processing diagram.

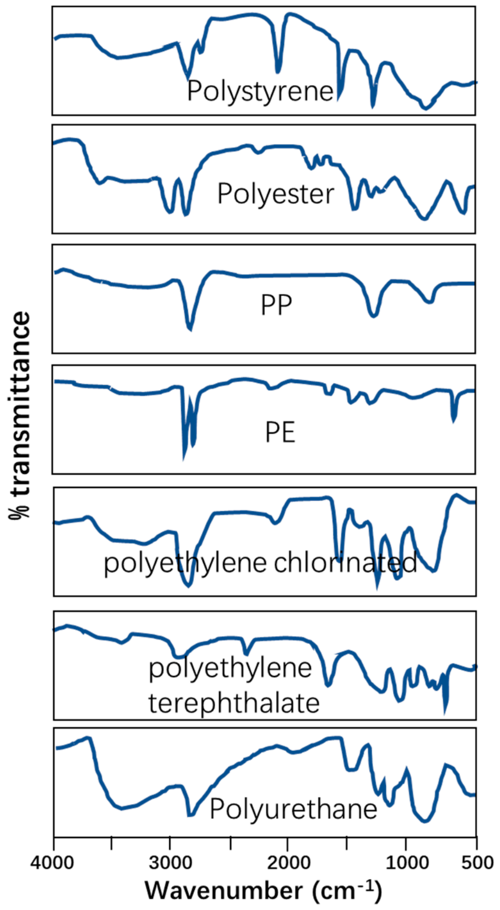

Figure S4. Typical FT-IR spectra of the polymers of MPs detected in TOFCs.
